# Protective effects of platelet-rich plasma against lidocaine cytotoxicity on canine articular chondrocytes

**DOI:** 10.1186/s13028-018-0418-0

**Published:** 2018-10-26

**Authors:** Erika Bianchini, Francesco Mancini, Antonio Di Meo, Anna Stabile, Sandra Buratta, Livia Moscati, Alessandra Pistilli, Claudia Floridi, Marco Pepe, Elisabetta Chiaradia

**Affiliations:** 1 Department of Veterinary Medicine, University of Perugia, Perugia, Italy; 2 Department of Surgery and Biomedical Sciences, University of Perugia, Perugia, Italy; 3 Department of Chemistry, Biology and Biotechnology, University of Perugia, Perugia, Italy; 4 Istituto Zooprofilattico Sperimentale dell’Umbria e della Marche, Perugia, Italy

**Keywords:** Apoptosis, Cell viability, Chondrocytes, Dog, Lidocaine, Platelet-rich plasma

## Abstract

**Background:**

Lidocaine (LD) is one of the most commonly used local anesthetics for performing arthroscopic surgery and managing of osteoarthritic pain in both human and veterinary medicine. However, over the last years, several studies have focused on the chondrotoxic effects of LD. In order to ensure that intra-articular lidocaine is safe to use, treatments aimed at mitigating chondrocyte death have recently been investigated. The aim of this study is to evaluate the possible protective effects of platelet-rich plasma (PRP) against LD cytotoxicity on canine articular chondrocytes.

**Results:**

Articular canine chondrocytes, were exposed to 1% or 1.8% LD alone or in co-presence with 10% PRP for 30 min. In order to evaluate the effects of PRP pre-treatments, experiments were carried out on cells cultured in serum-free medium-or in medium supplemented with 10% PRP or 10% fetal bovine serum. Cell viability was evaluated by methyl thiazolyl tetrazolium assay and cell apoptosis was analyzed by flow cytometry using annexin V-fluorescein isothiocyanate/propidium iodide. The results showed that LD significantly reduced canine chondrocytes viability, probably due to apoptosis induction. Pre-treatment or the co-presence of PRP in the media restored the number of viable chondrocytes. The PRP also seemed to protect the cells from LD-induced apoptosis.

**Conclusions:**

Pre-treatments and/or the simultaneous administration of PRP reduced LD-induced cytotoxicity in canine chondrocytes. Further in vivo studies are required to determine whether PRP can be used as a save protective treatment for dogs receiving intra-articular LD injections.

## Background

Lidocaine (LD) is one of the most commonly used local anesthetics for pre and post-operative veterinary joint surgery and chronic osteoarthritic pain management [1]. Local anesthetics reduce pain without altering brain or breathing functions. Thus, intra-articular (IA) local anesthetic injections now routinely used instead of general anesthesia, reduce the systemic effects of drugs. Indeed, they act locally on joint pain lowering the risk of adverse effects on the central nervous gastrointestinal, respiratory, and cardiovascular systems [1, 2]. However, there has been much debate on using LD as local anesthetic due to its in vitro and in vivo chondrotoxic effects [3–7]. In particular, LD proved to be cytotoxic to bovine [4, 8], equine [5], human [9, 10] and canine [11] articular chondrocytes. The underlying mechanisms for these chondrotoxic effects have not yet been established, but apoptosis and mitochondrial damage have been suggested in human chondrocytes [7, 12, 13].

In order to ensure the safe use of IA LD, some treatments aimed at mitigating cell death have recently been investigated. In particular, some authors have proposed the co-administration of *N*-acetyl cysteine [14] or hyaluronic acid [15, 16], for reducing the local anesthetics toxicity and as healing-promoters.

In the last decades, platelet-rich plasma (PRP) has been used for improving clinical symptoms of joint disorders, especially in long-lasting orthopaedic pathologies like osteoarthritis [17–20]. PRP is a blood derivate with a higher concentration of platelets which is rich in growth-factors such as transforming growth factor (TGF), platelet-derived growth factor (PDGF), insulin growth factor (IGF) and others [21]. In particular, PRP has antiinflammatory, anabolic and differentiation potential for slow-growing cell-populations such as tenocytes or chondrocytes [18, 19, 22]. The PRP has been proved effective in the treatment of different musculoskeletal injuries [23, 24]. Moreover, it has been observed that PRP protects human tenocytes against cell death induced by dexamethasone and ciprofloxacin [25]. The potential protective effect of PRP on human chondrocyte in vitro against LD and corticosteroids has recently been reported [26]. To the authors’ knowledge, to date, no studies have been carried out using PRP to protect canine chondrocytes against the toxic effects of LD.

The aim of this study was to evaluate the effects of LD and PRP co-treatment on canine articular chondrocytes. The cells were exposed to toxic concentrations of LD (1–1.8%) with or without PRP. The study was based on the hypothesis that the pro-healing effect of PRP can protect canine chondrocytes against LD-induced cell-death. The purpose of the study was to open new possibilities in the field of veterinary medicine, by administering PRP and LD in a single intra-articular injection for managing perioperative and chronic joint pain without risk of cartilage damage.

## Methods

### Primary cultures of canine chondrocytes

Chondrocytes were isolated from healthy articular cartilage of the knee/tarsal joints of five dogs (4–8 years old) of different breeds. All of the dogs used for tissues collection had died or been euthanized for reasons unrelated to this study or musco-skeletal pathologies. All experimental practices were approved by the University’s ethical committee (no. 2015-004). Following aseptic skin preparation with chlorhexidine gluconate, the joint specimens, obtained within 1–2 h after death, were disarticulated. Cartilage slices were harvested under sterile conditions, using sterile scalpels, rinsed twice in Dulbecco’s phosphate-buffered saline (DPBS) (Sigma-Aldrich, Saint Louis, USA), without Ca^2+^ and Mg^2+^, containing 100 U/mL of penicillin, 100 µg/mL of streptomycin and 250 μg/mL amphotericin B, and minced. The slices were then digested primarily with 0.25% trypsin for 10 min at 37 °C and subsequently with 2 mg/mL collagenase type IA (Sigma-Aldrich) at 37 °C for 6–8 h. Undigested tissue was separated from cells using a 70-μm cell strainer (Becton–Dickinson, Franklin Lakes, USA). Cells were collected by centrifugation (10 min at 700×*g*), washed in DPBS, re-suspended in Dulbecco’s modified Eagle’s medium (DMEM) (Sigma-Aldrich) and supplemented with 10% fetal bovine serum (FBS) (Sigma-Aldrich), 100 U/mL of penicillin and 100 µg/mL of streptomycin. Cells at a density of 10 × 10^3^/cm^2^ were then seeded in culture flasks and expanded in a monolayer at 37 °C in a humidified atmosphere of 5% CO_2_. When the monolayers reached confluence, the cells were detached enzymatically from the flask with 0.25% trypsin–EDTA (Sigma-Aldrich) and subcultured. Chondrocytes of the second passage of subculture were used for all experiments with the aim of minimizing phenotype drift.

### PRP preparation

PRP was prepared with whole blood collected from the jugular vein in citrate-dextrose solution vacutainer (Becton–Dickinson-Vacutainer^®^) by using double centrifuge method. Briefly, the blood samples were firstly centrifuged at 200×*g* for 20 min at 25 °C (Centrifuge 5810 R—Eppendorf, Hamburg, Germany), the upper layer, containing platelets and a few WBCs, was then collected and centrifuged at 1800×*g* for 10 min at 25 °C in order to separate platelet (PLT) pellet from platelet poor plasma (PPP). The platelet pellet was resuspended in a volume of PPP at final concentration of 2.0 × 10^6^ PLT/µL and the excess PPP was discarded. The PRP used in our experiments had final leukocyte (WBC) and erythrocyte (RBC) concentrations of 0.678 ± 0.43 10^9^/L and 0.206 ± 0.21510^12^/L respectively. PLT, WBC and RBC counts were determined with a hemocytometer (EosBIO, Cervarese Santa Croce, Italy).

In accordance with previous studies [17–19, 25] 10% PRP was used for all the experiments.

### Assessment of chondrocyte viability

The effects of both PRP and LD on canine chondrocyte viability were assessed using methyl thiazolyl tetrazolium (MTT) assay (Sigma Aldrich) based on the mitochondrial dehydrogenase activity of living cells. Briefly, the cells were seeded, in triplicate in the 96-well plates, at a density of 15 × 10^3^ cells/well and cultured at 37 °C in a humidified atmosphere of 5% CO_2_ for 24 h. The cells were then divided into three experimental groups and maintained in medium alone (starved-cells), in medium supplemented with 10% FBS (FBS-cells) or in medium supplemented with 10% PRP (PRP-cells). After 24 h, the chondrocytes were treated with final concentrations of 1% and 1.8% of LD (S.A.L.F., Bergamo. Italy) alone or in association with 10% PRP for 30 min. Dilutions of LD and PRP were prepared in DPBS. Although the most commonly administered doses of LD are 1% and 2% [27] we used 1.8% instead 2% in order to obtain 10% of PRP in association with LD, as the highest commercially available concentration of LD is at 2%.

At the end of treatments, medium was discarded and a new medium containing 20 μL of MTT solution (5 mg/mL) was added to each well. After 4 h, the resulting purple formazan crystals were solubilised in dimethyl sulfoxide and the absorbance was determined at 570 nm (with correction of absorbance at 620 nm) using a Multiskan GO Microplate Spectrophotometer (Thermo Fisher Scientific—Waltham, USA). Cell viability was expressed as the percentage of treated cells compared with untreated starved-cell, assuming that the absorbance of these cells was 100%.

### Assessment of apoptosis by flow cytometric analyses

**Fig. 1:**
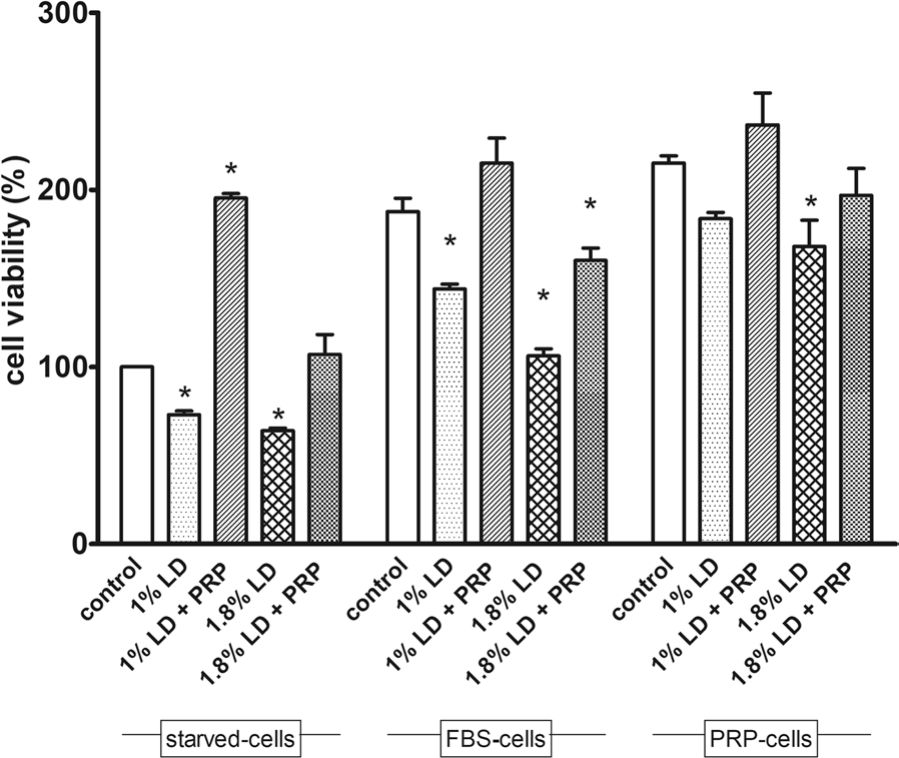
Cell viability. The effects of LD alone or with 10% PRP on cell viability were assayed in chondrocytes maintained for 24 h in medium (starved-cells), in medium containing 10% FBS (FBS-cell) or 10% PRP (PRP-cells). Date are expressed as mean ± SD. The experiments were repeated thrice, in triplicates. *P < 0.005 vs cell untreated of each experimental group (PBS)

Apoptosis was assessed by Annexin V-fluorescein isothiocyanate/propidium iodide (annexin V-FITC/PI) binding kit (Becton–Dickinson) using flow cytometry. To this aim, the cells were seeded at 4 × 10^4^ cells/cm^2^ in 6-well plates and incubated for 24 h at 37 °C in a humidified atmosphere of 5% CO_2_. The cells, were then maintained in standard conditions (medium supplemented with 10% FBS) for 24 h, after which the cells were exposed to 1% LD with or without 10% PRP for 30 min (Fig. 1). After the treatments, the mediums were collected and the cells were detached with 0.05% of DPBS/EDTA. The chondrocytes were washed twice with DPBS without Ca^2+^ and Mg^2+^. Procedures for labelling of of Annexin V-FITC/PI were carried out according to the manufacturers’ instructions. Flow cytometry data acquisition was performed on a FACSCalibur platform (Becton–Dickinson) equipped with 488 and 633 nm lasers and running CellQuest Software (Becton–Dickinson).

### Statistical analysis

Data were presented as mean ± standard deviation (SD). Oneway analysis of variance (ANOVA) and Bonferroni post hoc test were performed for statistical analysis. A P value less than 0.05 was considered statistically significant. Each experiment was performed in triplicate and repeated at least three times.

## Results

### Effects of lidocaine and PRP on canine chondrocyte viability

Cell viability of the three experimental groups is expressed as percentage compared to the control and it is reported in Fig. 1.

**Fig. 2:**
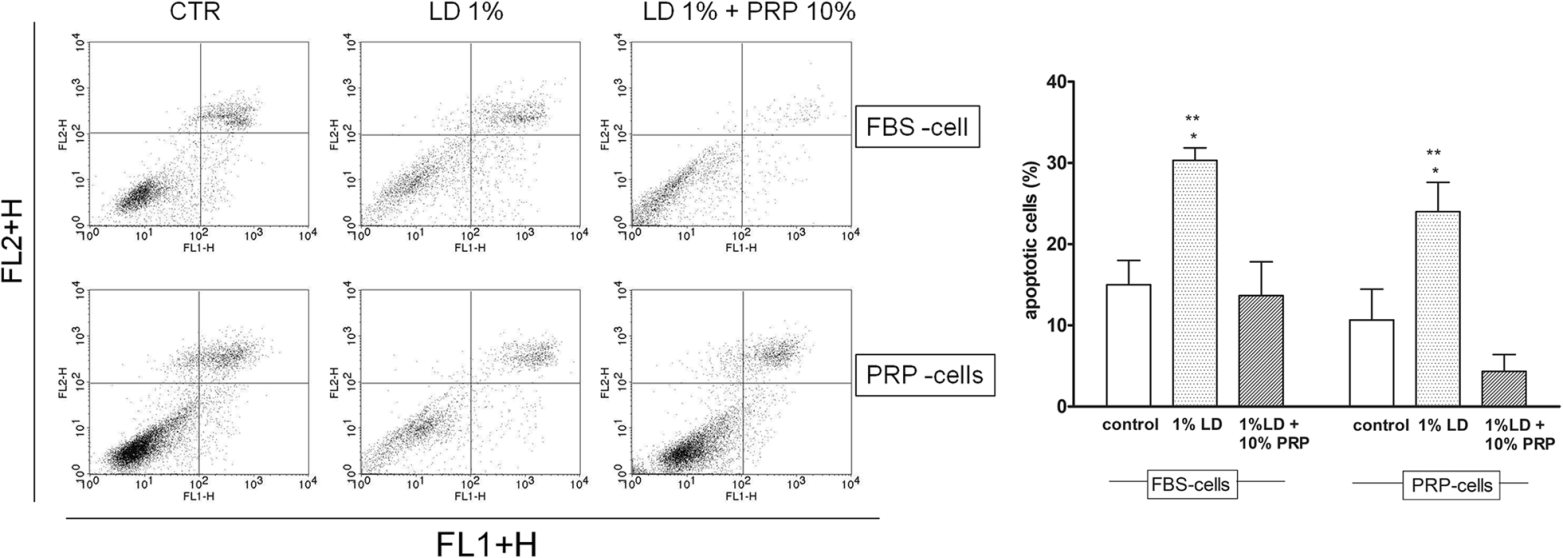
Flow cytometry analysis by Ann V/PI assay for apoptosis. Chondrocytes were cultured for 24 h in medium with 10% FBS (FBS-cells) or in medium with 10% PRP (PRP-cells) and then treated with 1% LD, 1% LD + 10% PRP or 10%PBS. **a** Representative dot plots of the two experimental groups, are shown. Different subpopulations were classified in necrotic cells (V−/PI+), late apoptotic cells (V+/PI+), live cells (V−/PI−) and early apoptotic cells (V+/PI−). **b** Bar graphs represent the percentage (mean ± SD) of apoptotic cells (early + late apoptotic cells) in each condition. The experiments were repeated thrice, in triplicates *P < 0.05 1% LD vs PBS in each experimental group; **P < 0.001 1% LD vs 1% LD + 10% PRP in each experimental group

PRP was able to protect the starved cells from the cytotoxic effect of both 1% or 1.8% LD. Surprisingly a significant increase in cell viability compared to CTR cells (P < 0.001) was observed when the cells were exposed to 10% PRP for only 30 min, even in co-presence of 1% LD. LD induced a statistically significant reduction in cell viability in FBS-cells that reached 43% for 1.8% LD (P < 0.01). 10% PRP restored cell viability when added to a medium containing 1% LD but it did not restore completely the viability of cells treated with 1.8% LD, even though PRP also proved to be partially protective (Fig. 2).

Only 1.8% LD was able to induce a significant reduction in cell viability (22%), in PRP-cells, compared to the cells treated with PBS (P < 0.035). However, no significant differences were found among other comparisons in this experimental group.

### Effects of lidocaine and lidocaine/PRP on canine chondrocyte apoptosis

Cytofluorimetric analysis of the PRP effects on LD-induced chondrotoxicity was carried out on cells exposed to 1% LD.

The FACS analysis showed a protective effect of PRP LD-induced apoptosis (Fig. 2). LD 1% caused an increase in the percentage of late-apoptotic cells in both experimental groups. These effects were reduced by co-administration with PRP. More specifically, the percentage of apoptotic cells were 15 ± 3, 30.3 ± 1.52 and 13.66 ± 4.16 in PBS, 1% LD and 1% LD + 10% PRP in control cells, and 10.66 ± 3.78, 24 ± 3.60 and 4.33 ± 2.016 in PRP-cells respectively. LD did not cause necrosis in both experimental groups.

## Discussion

This study investigates the effects of PRP on LD-induced cytotoxicity in canine in vitro chondrocytes. LD is one of the most commonly used local anesthetics for managing post-operative IA pain following arthroscopy. Previous in vitro and in vivo studies have investigated the potential toxic effects of this local anaesthetic on chondrocytes [6, 28–31]. However, arthroscopic surgery as well as IA local anesthetic treatments, have become routine procedures in veterinary medicine.

Therefore, carrying out simultaneous pain management and healing therapies represents a challenge for orthopaedic practitioners. Of all the regenerative joint treatments, promising results have been obtained with different formulation of PRP in vivo [32, 33]. Several studies have shown the proliferative potential of PRP on chondrocytes for various species (porcine, human, bovine, ovine, equine) [19, 24, 34]. PRP deemed to be a promising cartilage repair treatment and it can be used as a bioactive scaffold with anabolic and antiinflammatory properties [19, 35]. Therefore, this autologous preparation may prove to be useful for counteracting noxious effects of local anesthetics thus creating a safe joint healing environment. Although some papers report the adverse effects of blood or its components such as platelets [36], fibrin(ogen) [37], complement [38] on joint tissues, the positive outcomes obtained with PRP treatments has been widely documented [17–20, 22–24]. Our results are in accordance with Durant et al. that shows the protective effect of PRP against 1% and 0.5% LD bupivacaine on human chondrocytes [26]. Moreover, our results demonstrate that PRP is also able to protect chondrocytes at the concentration of 1.8% which is close to the maximum recommended in vivo dose (2%) [27], and shows that PRP may reduce LD-induced apoptosis. Baboldashti et al. [25] previously demonstrated that PRP protects tenocytes from the adverse effects of dexamethasone and ciprofloxacin, and proposed using PRP to minimize the effect of unsafe therapeutic treatments. However, the co-administration of hyaluronic acid with local anesthetics such as LD and bupivacaine was only effective in reducing the chondrotoxicity of bupivacaine but not of LD [14].

We herewith confirm the dose-dependent toxic effects of LD on canine chondrocytes reported by Di Salvo et al. [11] and demonstrate the proliferative effects of 10% PRP on canine chondrocytes, which to our knowledge have not previously been investigated. Our results indicate that PRP is able to restore chondrocytes viability affected by LD. The co-treatment of PRP and LD (1% or 1.8%) enhances chondrocytes viability in starved cells, respect to drug alone. In these cells, PRP elicits a highly proliferative effects even if in co-presence with 1% LD, which suggests that at this concentration, LD is unable to overcome PRP proliferative stimuli. In PRP-cells, only 1.8% LD causes a significant decrease in cell viability and co-treatment with PRP is able to restore cell viability to control (cell treated with PBS) values. Contrastingly, in FBS-cells, the reduction of cell viability is observed at both LD concentrations (1% or 1.8%) and PRP was able to restore the values of the cells treated with PBS only in co-presence with 1% LD. Moreover, FBS-cells are more susceptible to 1.8% LD than PRP-cells. These results clearly indicate that PRP is able to prevent and/or counteract the adverse effects of LD even at clinically-relevant concentrations. We can exclude that the protective effects of PRP is due to its nutrient content, because no protection was observed in FBS-cells, maintained in medium supplemented with FBS which contains required nutrient and other necessary substances to sustained cell growth. Other treatments like adrenaline, proved to be ineffective against the detrimental effects of 2% LD yet, was protective against LD-induced cell death when co-administered with 1% LD [11]. *N*-acetyl cysteine (NAC) [15] and hyaluronic acid were found to be cytoprotective in human chondrocytes [16] even if the LD concentrations tested were lower than those commonly used in clinical practice.

This study showed that LD reduces viable cell percentage in canine chondrocytes by inducing apoptosis. Similar effects have been reported in chondrocytes from other species [5, 6, 15, 16] and other cell types [39, 40].

We also demonstrated that PRP exerts a protective action against LD-induced apoptosis. The pro-apoptotic effect exerted by LD is nullified by the co-presence of PRP.

Over all our results suggest that PRP elicits protective effects against LD-induced chondrotoxicity, by stimulating both anti-apoptotic and proliferating pathways. Most of the growth factors (GFs) of PRP have been described as proliferation and apoptosis modulators [41, 42]. For example, IGF is a protein involved in cell growth and regeneration [43], and its down-regulation is associated with an increase in apoptosis. Another important GF in PRP is PDGF, which is a negative regulator of apoptosis, either alone or in synergy with IGF [44–47]. The protective effects of PRP against LD-induced apoptosis may also be due to its ability to inhibit the cleavage of caspase 3 [43] which appears to be induced by LD [13, 48, 49]. Mitogen-activated protein kinase pathway [50], caspase 3-mediated apoptosis [15, 16, 49] and p53-dependent mitochondrial apoptotic pathway [16] are deemed to be the molecular mechanisms of LD-induced chondrocytes death. Future research should address the cytosolic signaling pathways involved in PRP-chondroprotection; in vivo studies are also required to evaluate co-injection of PRP and LD.

This is an in vitro study, and therefore its main limitation is that it does not consider the condition and the biology of joint during the post-operative period or when inflammatory conditions occur. However, the suggested co-administration of LD and PRP in vivo may be used to improve joint pain management. This is because PRP could act as a modulating nuclear factor-κB signaling pathway, by alleviating inflammation and angiogenesis, thus reducing the pain associated with the pain receptors located on the synovial membrane. Moreover, there are other factors that may potentiate the effects of PRP and limit the toxic effects of anesthetics in vivo [51]. In fact, synovial fluid could represent a dilutor for LD thus reducing its dosage and consequently its detrimental toxic effects [52]. However, although dilution can also occur for PRP, the joint modified by surgery or arthrosis could become a “platelet-activator”, thus stimulating platelet degranulation [53].

## Conclusions

Local anaesthetics have been found to exert toxic effects on articular chondrocytes even if they are commonly used for arthroscopic surgery and arthritis pain management. Therefore, it is essential to find alternative treatments capable of minimizing their adverse effects on chondrocytes.

This study demonstrates that previous and/or the simultaneous administration of PRP could prevent the toxic effects of LD, such as the reduction in cell viability and apoptosis, in canine chondrocytes in vitro. Therefore, PRP may prove to be an effective protective treatment for dogs injected with LD for the management of perioperative and chronic joint pain. However, further in vivo studies are needed to confirm the safety of this possible pain management practice.

## Abbreviations

Annexin V-FITC/PI: annexin V-fluorescein isothiocyanate/propidium iodide
ANOVA: analysis of variance
DPBS: Dulbecco’s phosphate-buffered saline
DMEM: Dulbecco’s modified Eagle’s medium
FBS: foetal bovine serum
GFs: growth factors
hrs: hours
IA: intra-articular
IGF: insulin-like growth factor
L: liter
LD: lidocaine
min: minutes
MTT: methyl thiazolyl tetrazolium
PDGF: platelet derived growth factor
PLT: platelet
PPP: platelet-poor plasma
PRP: platelet-rich plasma
RBC: red blood cell
SD: standard deviation
TGF: transforming growth factor
WBC: leukocyte

## References

[1] McLure HA, Rubin AP (2005). Review of local anaesthetic agents. Minerva Anestesiol.

[2] Pyati S, Gan TJ (2007). Perioperative pain management. CNS Drugs.

[3] Hepburn J, Walsh P, Mulhall KJ (2011). The chondrotoxicity of local anaesthetics: any clinical impact?. Joint Bone Spine.

[4] Miyazaki T, Kobayashi S, Takeno K, Yayama T, Meir A, Baba H (2011). Lidocaine cytotoxicity to the bovine articular chondrocytes in vitro: changes in cell viability and proteoglycan metabolism. Knee Surg Sports Traumatol Arthrosc.

[5] Park J, Sutradhar BC, Hong G, Choi SH, Kim G (2011). Comparison of the cytotoxic effects of bupivacaine, lidocaine, and mepivacaine in equine articular chondrocytes. Vet Anaesth Analg.

[6] Baker JF, Mulhall KJ (2012). Local anaesthetics and chondrotoxicity: what is the evidence?. Arthroscopy.

[7] Gulihar A, Robatib S, Twaijc H, Salihb A, Taylord GJS (2015). Articular cartilage and local anaesthetic: a systematic review of the current literature. J Orthop.

[8] Karpie JC, Chu CR (2007). Lidocaine exhibits dose- and time-dependent cytotoxic effects on bovine articular chondrocytes in vitro. Am J Sports Med.

[9] Jacobs TF, Vansintjan PS, Roels N, Herregods SS, Verbruggen G, Herregods LL (2011). The effect of lidocaine on the viability of cultivated mature human cells: an in vitro study. Knee Surg Sports Traumatol Arthrosc.

[10] Dragoo JL, Braun HJ, Kim HJ, Phan HD, Golish SR (2012). The in vitro chondrotoxicity of single-dose local anesthetics. Am J Sports Med.

[11] Di Salvo A, Chiaradia E, della Rocca G, Mancini F, Galarini R, Giusepponi D (2016). Intra-articular administration of lidocaine plus adrenaline in dogs: pharmacokinetic profile and evaluation of toxicity in vivo and in vitro. Vet J.

[12] Johnson ME, Uhl CB, Spittler KH, Wang H, Gores GJ (2004). Mitochondrial injury and caspase activation by the local anesthetic lidocaine. Anesthesiology.

[13] Grishko V, Xu M, Wilson G, Pearsall AW (2010). Apoptosis and mitochondrial dysfunction in human chondrocytes following exposure to lidocaine, bupivacaine, and ropivacaine. J Bone Joint Surg Am.

[14] Kim R, Kang JR, Hah YS, Park HB (2017). *N*-acetyl cysteine protects cells from chondrocyte death induced by local anesthetics. J Orthop Res.

[15] Onur TS, Sitron CS, Dang A (2013). Co-administration of hyaluronic acid with local anaesthetics shows lower cytotoxicity than local anaesthetic treatment alone in bovine articular chondrocytes. Bone Joint Res.

[16] Lee YJ, Kim SA, Lee SH (2016). Hyaluronan suppresses lidocaine-induced apoptosis of human chondrocytes in vitro by inhibiting the p53-dependent mitochondrial apoptotic pathway. Acta Pharmacol Sin.

[17] Everts PAM, Knape JTA, Weibrich G, Schönberger J, Hoffmann J, Overdevest EP (2006). Platelet-rich plasma and platelet gel: a review. J Extra Corpor Technol.

[18] Zhu Y, Yuan M, Meng HY, Wang AY, Guo QY, Wang Y (2013). Basic science and clinical application of platelet-rich plasma for cartilage defects and osteoarthritis: a review. Osteoarthritis Cartilage.

[19] Xie X, Zhang C, Tuan RS (2014). Biology of platelet-rich plasma and its clinical application in cartilage repair. Arthritis Res Ther.

[20] Marmotti A, Rossi R, Castoldi F, Roveda E, Michielon G, Peretti GM (2015). PRP and articular cartilage: a clinical update volume. Biomed Res Int.

[21] Marx RE (2001). Platelet-rich plasma (PRP): what is PRP and what is not PRP?. Implant Dent.

[22] Sakata R, Reddi AH (2016). Platelet-rich plasma modulates actions on articular cartilage lubrication and regeneration. Tissue Eng Part B Rev.

[23] Sampson S, Gerhardt M, Mandelbaum B (2008). Platelet rich plasma injection grafts for musculoskeletal injuries: a review. Curr Rev Musculoskelet Med.

[24] Brossi PM, Moreira JJ, Machado TS, Baccarin RY (2015). Platelet-rich plasma in orthopedic therapy: a comparative systematic review of clinical and experimental data in equine and human musculoskeletal lesions. BMC Vet Res.

[25] Baboldashti NZ, Poulsen RC, Franklin SL, Thompson MS, Hulley PA (2011). Platelet-rich plasma protects tenocytes from adverse side effects of dexamethasone and ciprofloxacin. Am J Sports Med.

[26] Durant TJS, Dwyer CR, McCarthy MBR, Cote MP, Bradley JP, Mazzocca AD (2017). Protective nature of platelet-rich plasma against chondrocyte death when combined with corticosteroids or local anesthetics. Am J Sports Med.

[27] Jacob B, Zippelius T, Kloss N, Benad K, Schwerdt C, Hoff P (2018). Local anesthetics’ toxicity toward human cultured chondrocytes: a comparative study between lidocaine, bupivacaine, and ropivacaine. Cartilage.

[28] Solomon DJ, Navaie M, Stedje-Larsen ET, Smith JC, Provencher MT (2009). Glenohumeral chondrolysis after arthroscopy: a systematic review of potential contributors and causal pathways. J Arthrosc Relat Surg.

[29] Busfield BT, Romero DM (2009). Pain pump use after shoulder arthroscopy as a cause of glenohumeral chondrolysis. Arthroscopy.

[30] Piper SL, Kramer JD, Kim HT, Feeley BT (2011). Effects of local anesthetics on articular cartilage. Am J Sports Med.

[31] Onur T, Dang A (2014). Reduction of environmental temperature mitigates local anesthetic cytotoxicity in bovine articular chondrocytes. J Sports Sci Med.

[32] Yin W, Qi X, Zhang Y, Sheng J, Xu Z, Tao S (2016). Advantages of pure platelet-rich plasma compared with leukocyte- and platelet-rich plasma in promoting repair of bone defects. J Transl Med.

[33] Arnoczky SP, Delos D, Rodeo SA (2011). What is platelet-rich plasma?. Oper Tech Sports Med.

[34] Carmona JU, Ríos DL, López C, Álvarez ME, Pérez JE, Bohórquez ME (2016). In vitro effects of platelet-rich gel supernatants on histology and chondrocyte apoptosis scores, hyaluronan release and gene expression of equine cartilage explants challenged with lipopolysaccharide. BMC Vet Res.

[35] Yin Z, Yang X, Jiang Y, Xing L, Xu Y, Lu Y (2014). Platelet-rich plasma combined with agarose as a bioactive scaffold to enhance cartilage repair: an in vitro study. J Biomater Appl.

[36] Boilard E, Blanco P, Nigrovic PA (2012). Platelets: active players in the pathogenesis of arthritis and SLE. Nat Rev Rheumatol.

[37] Flick MJ, LaJeunesse CM, Talmage KE, Witte DP, Palumbo JS, Pinkerton MD (2007). Fibrin(ogen) exacerbates inflammatory joint disease through a mechanism linked to the integrin αMβ2 binding motif. J Clin Invest.

[38] John T, Stahel PF, Morgan SJ, Schulze-Tanzil G (2007). Impact of the complement cascade on posttraumatic cartilage inflammation and degradation. Histol Histopathol.

[39] Honda H, Gotoh M, Kanazawa T, Nakamura H, Ohta K, Nakamura K (2016). Effects of lidocaine on torn rotator cuff tendons. J Orthop Res.

[40] Friederich P, Schmitz TP (2002). Lidocaine-induced cell death in a human model of neuronal apoptosis. Eur J Anaesthesiol.

[41] Anitua E, Sanchez M, Nurden AT, Zalduendo MM, De la Fuente M, Azofra J (2007). Platelet-released growth factors enhance the secretion of hyaluronic acid and induce hepatocyte growth factor production by synovial fibroblasts from arthritic patients. Rheumatology.

[42] Au L, Sashindranath M, Borg RJ, Kleifeld O, Andrews RK, Gardiner EE (2014). Activated platelets rescue apoptotic cells via paracrine activation of EGFR and DNA-dependent protein kinase. Cell Death Dis.

[43] Dimauro I, Grasso L, Fittipaldi S, Fantini C, Mercatelli N, Racca S (2014). Platelet-rich plasma and skeletal muscle healing: a molecular analysis of the early phases of the regeneration process in an experimental animal model. PLoS ONE.

[44] Bennett MR, Evan GI, Schwartz SM (1995). Apoptosis of human vascular smooth muscle cells derived from normal vessels and coronary atherosclerotic plaques. J Clin Invest.

[45] Stewart CE, Rotwein P (1996). Insulin-like growth factor-2 is an autocrine survival factor for differentiating myoblasts. J Biol Chem.

[46] Desai BJ, Gruber HE (1999). Anti-apoptotic actions of cytokines in mammalian cells. Proc Soc Exp Biol Med.

[47] Yi C, Ma C, Xie Z, Zhang G, Song W, Zhou X (2013). Down-regulation of programmed cell death 5 by insulin-like growth factor 1 in osteoarthritis chondrocytes. Int Orthop.

[48] Werdehausen R, Braun S, Essmann F, Schulze-Osthoff K, Walczak H, Lipfert P (2007). Lidocaine induces apoptosis via the mitochondrial pathway independently of death receptor signaling. Anesthesiology.

[49] Wohlrab D, Vocke M, Klapperstück T, Hein W (2005). The influence of lidocaine and verapamil on the proliferation, CD44 expression and apoptosis behavior of human chondrocytes. Int J Mol Med.

[50] Chang YC, Hsu YC, Liu CL, Huang SY, Hu MC, Cheng SP (2014). Local anesthetics induce apoptosis in human thyroid cancer cells through the mitogen activated protein kinase pathway. PLoS ONE.

[51] Bendinelli P, Matteucci E, Dogliotti G, Corsi MM, Banfi G, Maroni P (2010). Molecular basis of anti-inflammatory action of platelet-rich plasma on human chondrocytes: mechanisms of NF-kappaB inhibition via HGF. J Cell Physiol.

[52] Kamath R, Strichartz G, Rosenthal D (2008). Cartilage toxicity from local anesthetics. Skeletal Radiol.

[53] Andia I, Sánchez M, Maffulli N (2012). Joint pathology and platelet-rich plasma therapies. Expert Opin Biol Ther.

